# Snowflake epitope matching correlates with child-specific antibodies during pregnancy and donor-specific antibodies after kidney transplantation

**DOI:** 10.3389/fimmu.2022.1005601

**Published:** 2022-10-28

**Authors:** Matthias Niemann, Yara Strehler, Nils Lachmann, Fabian Halleck, Klemens Budde, Gideon Hönger, Stefan Schaub, Benedict M. Matern, Eric Spierings

**Affiliations:** ^1^ Research and Development, PIRCHE AG, Berlin, Germany; ^2^ Center for Tumor Medicine, H&I Laboratory, Charité University Medicine Berlin, Berlin, Germany; ^3^ Department of Nephrology and Medical Intensive Care, Charité-Universitätsmedizin Berlin, Berlin, Germany; ^4^ Clinic for Transplantation Immunology and Nephrology, University Hospital Basel, Basel, Switzerland; ^5^ Transplantation Immunology, Department of Biomedicine, University of Basel, Basel, Switzerland; ^6^ HLA-Diagnostics and Immunogenetics, Department of Laboratory Medicine, University Hospital Basel, Basel, Switzerland; ^7^ Center for Translational Immunology, University Medical Center, Utrecht, Netherlands; ^8^ Central Diagnostic Laboratory, University Medical Center, Utrecht, Netherlands

**Keywords:** HLA, epitope matching, solvent accessibility, deep learning, kidney transplantation, pregnancy

## Abstract

Development of donor-specific human leukocyte antigen (HLA) antibodies (DSA) remains a major risk factor for graft loss following organ transplantation, where DSA are directed towards patches on the three-dimensional structure of the respective organ donor’s HLA proteins. Matching donors and recipients based on HLA epitopes appears beneficial for the avoidance of DSA. Defining surface epitopes however remains challenging and the concepts underlying their characterization are not fully understood. Based on our recently implemented computational deep learning pipeline to define HLA Class I protein-specific surface residues, we hypothesized a correlation between the number of HLA protein-specific solvent-accessible interlocus amino acid mismatches (arbitrarily called Snowflake) and the incidence of DSA. To validate our hypothesis, we considered two cohorts simultaneously. The kidney transplant cohort (KTC) considers 305 kidney-transplanted patients without DSA prior to transplantation. During the follow-up, HLA antibody screening was performed regularly to identify DSA. The pregnancy cohort (PC) considers 231 women without major sensitization events prior to pregnancy who gave live birth. Post-delivery serum was screened for HLA antibodies directed against the child’s inherited paternal haplotype (CSA). Based on the involved individuals’ HLA typings, the numbers of interlocus-mismatched antibody-verified eplets (AbvEPS), the T cell epitope PIRCHE-II model and Snowflake were calculated locus-specific (HLA-A, -B and -C), normalized and pooled. In both cohorts, Snowflake numbers were significantly elevated in recipients/mothers that developed DSA/CSA. Univariable regression revealed significant positive correlation between DSA/CSA and AbvEPS, PIRCHE-II and Snowflake. Snowflake numbers showed stronger correlation with numbers of AbvEPS compared to Snowflake numbers with PIRCHE-II. Our data shows correlation between Snowflake scores and the incidence of DSA after allo-immunization. Given both AbvEPS and Snowflake are B cell epitope models, their stronger correlation compared to PIRCHE-II and Snowflake appears plausible. Our data confirms that exploring solvent accessibility is a valuable approach for refining B cell epitope definitions.

## Introduction

As of today, kidney transplantation is the gold standard in renal replacement therapy. Amongst other factors, long-term graft survival is known to be dependent on patient and donor human leukocyte antigen (HLA) compatibility. HLA incompatible donor organs are recognized and affected by the recipient’s immune system - even under immunosuppressive therapy - eventually resulting in premature allograft loss (1–3). The definition of HLA compatibility remains challenging as not each amino acid difference between the HLA proteins of patient and donor represents an equal immunologic risk. Consequently several approaches have been proposed to refine the definition of histocompatibility from antigenic/allelic level to a functional level, each bearing its own challenges (4–6). Considering the HLA protein’s surface, the HLA Matchmaker model defines epitopes as antibody-accessible, potentially discontinuous patches of spatially close polymorphic amino acid residues (7). The EPRegistry database (epregistry.com.br) is a publicly accessible list of these so-called Eplets (8, 9). Not all of these epitopes are however unambiguously confirmed in antibody responses (10). The approach of counting the number of mismatched eplets (i.e. eplet/epitope load) introduces the assumption of equi-immunogenic eplets, which has been disproven (11, 12). The varying physicochemical properties of mismatched amino acids may be an explanation and matching amino acids based on their electrostatic potentials was shown to be advantageous over the unweighted eplet load (13). Focusing on the HLA protein conformation, the recently described HLA-EMMA algorithm considers amino acid mismatches at shared surface-accessible positions as potential B cell targets (14). HLAMatchmaker assumes two proteins with the same amino acid configuration as both carrying the same eplet, assuming identical folding or alleles within the same locus (15). Likewise, HLA-EMMA considers surface-accessible amino acid positions per HLA locus, which however may be not identical across different HLA proteins given slight variations in protein folding (16). Refining that concept, the proposed Snowflake algorithm defines the HLA protein-specific surface accessibility, taking into account structural deviations between HLA proteins. Amino acid mismatches at solvent accessible positions of the respective donor HLA are hypothesized to increase risk of B cell allorecognition (16). Within this work, we evaluated the correlation between the number of such HLA Class I-specific solvent-accessible amino acid mismatches (Snowflake score) and the incidence of donor-specific HLA antibodies (DSA) after kidney transplantation and child-specific HLA antibodies (CSA) during pregnancy, respectively (collectively immunizer-specific antibodies, ISA). For reference, we compared the Snowflake score to Eplet scores and PIRCHE-II scores. The considered algorithms were only applied to HLA-mismatched cases, allowing to evaluate value added to “classic” HLA-matching. We hypothesized, (i) Snowflake scores are higher in ISA-positive patients and (ii) that Snowflake scores correlate well with Eplet matching, given they both aim on defining B cell epitopes by HLA protein structure analysis.

## Method

### Pregnancy cohort

The previously described pregnancy cohort (PC) of the University Hospital Basel consists of 231 pregnant women who gave live birth between September 2009 and April 2011 (11, 17–19). The study was approved by the local ethics committee. The median age of the mothers was 31 years (Q1 = 28, Q3 = 35). Prior immunization events (blood transfusions, transplantations, or miscarriages) were ruled out. High-resolution typing for HLA-A, -B, -C, -DPA1, -DPB1, -DQA1, -DQB1, -DRB1, and -DRB3/4/5 was available for all study participants (i.e. women and children) by means of next-generation HLA sequencing (NGSgo^®^ HLA amplification and library preparation (www.gendx.com, GenDx, Utrecht, the Netherlands), sequencing on Illumina^®^ MiSeq™ (www.illumina.com, Illumina, San Diego, USA)). Maternal HLA antibody specificities were assessed previously, by single antigen beads (LabScreen™ Single HLA Antigen Beads (SAB), OneLambda Thermo Fisher, Canoga Park, CA, USA)) on sera collected between days 1 and 4 after delivery. Antibody data were mapped to the child HLA typing to identify CSA, considering a sample-specific biological cutoff of MFI values above 100 and exceeding the mean of all SAB_self-HLA mother_+ 3 SDs as reported previously (11).

### Kidney transplant cohort

The kidney transplant cohort (KTC) of the Charité University Hospital consists of 305 patients who underwent kidney transplantation between 2000 and 2019. The study was approved by the institutional review boards of the Charité hospital. HLA typing of the recipient was prospectively obtained by serological (HLA Class I) and DNA-based techniques (HLA Classes I and II). Serological typing was done by using the commercially available serological HLA typing trays HLA-Ready Plate ABC 144 (inno-train, Kronberg, Germany). DNA-based typing was achieved by using sequence-specific primer (SSP) (Olerup, Stockholm, Sweden) and reverse sequence-specific oligonucleotide (SSO) (One Lambda Thermo Fisher, Canoga Park, CA). All assays were performed according to the manufacturer’s instructions. Following local regulations, recipients were typed twice by the transplant center. HLA typing of deceased donors was provided by the donor center and confirmed by SSO in the transplant center. Living donors were typed on two occasions in the transplant center by serology and SSO or SSP. HLA typing data were collected on an intermediate resolution level allowing the assignment of serological equivalents.

Patients were regularly screened for the presence and identification of HLA antibodies prior to transplantation and during their follow-up at least on an annual schedule (Luminex^®^-based LABScreen™ mixed and SAB assay, OneLambda Thermo Fisher, Canoga Park, CA). HLA antibody specificities were mapped to kidney donor HLA typings to identify DSA. Enrolled patients did not have DSA prior to transplantation (i.e. MFI exceeding 1000) and had a negative complement-dependent-cytotoxicity crossmatch using unseparated as well as isolated T and B lymphocytes.

The long observation period allowed to consider all applied donor allocation schemes equally, the cohort comprises four matched groups: (1) highly sensitized patients that have been transplanted *via* the Acceptable Mismatch (AM) allocation program of Eurotransplant (94 patients), (2) Eurotransplant Kidney Allocation System (ETKAS)-allocated patients transplanted with a reported panel-reactive antibody level (PRA) of ≤ 5%(92 patients), (3) ETKAS-allocated patients transplanted with a reported PRA of >5% and < 85% (87 patients), and (4) highly-sensitized patients with PRA levels ≥ 85% allocated through ETKAS (32 patients). Additional demographic data on the KTC is provided in **Table 1**. In the full kidney transplant cohort of the Charité University Hospital, both B cell and T cell epitope matching algorithms were previously shown to correlate with DSA development and graft survival (20). Notably, the considered cohort was allocated *via* ETKAS and AM, both optimizing for the number of HLA-A-B-DR mismatches, which leads to a significantly lower average HLA-A-B and HLA-A-B-DR number of mismatches compared to reported US data (21). Although the allocation algorithms have not specifically matched for HLA-C and -DQB1, pre-formed donor-specific HLA antibodies against all five loci (including HLA-C and -DQB1) were considered as contraindication for donor offers.

**Table 1:** KTC characteristics and demographics.

Characteristic	Value
follow up, mean years, (SD)	6.6 (4.5)
recipient age at TX, mean years, (SD)	47.3 (12.2)
recipient male, number, (percentage)	164 (53.8%)
primary graft function, number, (percentage)	140 (45.9%)
cold ischemia time, hrs, (SD)	14.7 (4.8)
donor age at TX, mean years, (SD)	46.8 (13.5)
donor male, number, (percentage)	160 (52.5%)
waiting time, mean months, (SD)	56.3 (39.3)
antigen mismatches A-B, median, (IQR)	1 (1)
antigen mismatches A-B-DR, median, (IQR)	2 (1)
PRA at TX, median percentage, (IQR)	27% (41%)
peak PRA, median percentage, (IQR)	70% (48.5%)
blood group identical, number, (percentage)	45 (85.2%)

Antigen mismatches considering serologic split typing. SD, standard deviation; IQR, interquartile range.

### Snowflake epitope matching algorithm

The Snowflake algorithm is an integrated computational pipeline counting the number of HLA protein-specific solvent accessible amino acid mismatches between donor and recipient. It has been shown, solvent-accessibility is protein-specific, and thus proposed to be considered individually (16). For that purpose, Niemann et al. extracted 676 experimental HLA Class I structures of the Protein Data Bank (www.rcsb.org) and supplemented these by 37 predicted structures using the AlphaFold predictor (DeepMind Technologies Limited, London, UK) (22–24). Predicted structures have been augmented to 329 structures by adding known binding peptides into the HLA binding groove through docking simulation *via* the Anchored Peptide-MHC Ensemble Generator (APE-Gen) (25). The total set of structures covers all HLA Class I antigenic groups. Protein-residue-specific surface area was calculated considering the Shrake-Rupley algorithm (26). In order to extrapolate surface information to all known HLA Class I, a deep-learning neural network predictor was trained by considering amino acid sequence as input and surface area as output neurons. The predictor was used to create a comprehensive database of accessibility for all known HLA Class I proteins. This allowed solvent accessibility to be evaluated HLA protein-specifically, considering individual donor-HLA exposed amino acid positions to be matched with recipient HLA amino acid configurations. Comparisons with recipient HLA considered HLA-A, -B and -C simultaneously (i.e. interlocus). The full matching pipeline was implemented as a web service, featuring CSV input and output formats for batch processing (www.pirche.com, version 3.47) (16).

### Reference epitope matching algorithms

For comparison of the Snowflake algorithm with other epitope matching concepts within the clinical validation cohorts, the number of interlocus-mismatched donor eplets (AllEPS) and interlocus-mismatched antibody-verified eplets (AbvEPS) as defined by the HLA Epitope Registry (www.epregistry.com.br, version 3.0) was determined (9). Furthermore, the number of PIRCHE-II (www.pirche.com, version 3.47) was calculated (reviewed in (27)), considering the prediction of indirect T cell epitopes. In the PC, high resolution genotyping data on 11 loci allowed for PIRCHE analysis of HLA-DRB1 (i.e PIRCHE-II), -DRB3/4/5, -DQ and, -DP as independent donor-derived peptide presenters, as suggested before (19), yielding four separate PIRCHE matching scores.

### Imputation of HLA typing data in the KTC

The underlying core algorithms of all epitope matching methods ultimately require unambiguous protein-level HLA typing. To apply epitope matching using intermediate resolution level typings in the KTC, the previously described multiple imputation approach was refined (28). Multi allele codes were converted into antigen-specific candidate lists (https://hml.nmdp.org/MacUI/, IPD-IMGT/HLA version 3.47) (29). Given the rather homogenous cohort, potential haplotype pairs were fetched from the 2011 EURCAU NMDP haplotype dataset (30). The low-resolution-converted input typing-matched haplotype pairs were filtered by multi allele code candidate lists respectively. The remaining haplotype pairs’ frequencies were normalized by all remaining haplotype pairs. High-resolution pairs were only considered if the normalized frequency exceeds 1%. Epitope matching scores were calculated for each high-resolution recipient-donor-combination and summed up weighted by respective pair’s frequency. For PIRCHE, AllEPS, AbvEPS and Snowflake, this process is automated and integrated as a web-service (www.pirche.com).

### Pooling immunizer-specific antibody data

Snowflake score distributions in ISA-negative and ISA-positive cases were visualized by boxplots for each HLA locus individually. Regression analyses were carried out on pooled datasets of the respective cohort to increase sample size. To that extent, each mismatch of the PC and its corresponding CSA status was considered individually. Given the PC is haplotype-matched by design, each pregnancy translates into up to three data points in the pooled PC dataset, excluding homozygous and matched data points. Prior to aggregation, Snowflake, AllEPS and AbvEPS were centered by subtracting the respective mean and scaled by dividing the respective standard deviation to account for locus-specific ranges. PIRCHE scores were log-transformed based on previously reported logarithmic hazards (20). For the KTC, the respective locus’ epitope scores were normalized similarly. As organ donors may be mismatched on both HLA per locus, epitope scores were defined locus-specific rather than mismatch-specific. Correspondingly, the DSA status considers DSA against only one or both HLA of the donor as positive. Given low-resolution HLA typing data in the KTC, defining HLA mismatches is non-trivial, as allele-level mismatches may still occur in zero mismatch donor-recipient pairs. To still exclude the bias of HLA-matched data points, cases with zero PIRCHE, zero AbvEPS, zero AllEPS and zero Snowflake were excluded from analyses.

### Statistical analysis

In the pooled PC dataset, stepwise binomial logistic regression based on Akaike information criterion (31) was applied to evaluate independence of Snowflake and reference epitope matching algorithms. In the KTC dataset regression analyses were carried out by Cox proportional hazard and Cox multiple regression. Wilcoxon signed-rank test was applied to differences in epitope match score distributions between ISA-negative and ISA-positive cases. Correlation between epitope matching scores was analyzed by Spearman’s rank-correlation coefficient (Spearman’s Rho r_s_).

p-Values of less than 0.05 were considered statistically significant. Statistical calculations for the PC were executed in R software (R 4.2.0, R Foundation for Statistical Computing, Vienna, Austria), for the KTC in SPSS software (SPSS 27.0.0.0, IBM Corp., Armonk, NY).

## Results

**Figure 1:**
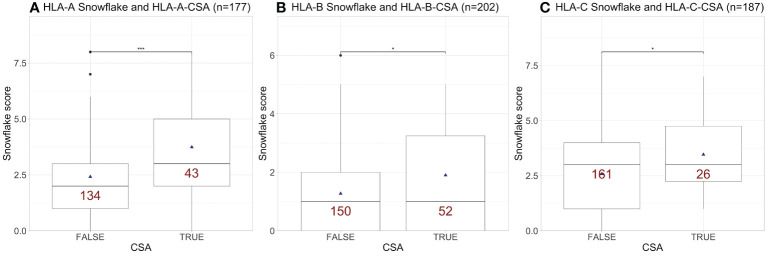
Snowflake score distribution of interlocus-restricted locus-specific HLA mismatches resulting in paternal mismatch-specific antibodies for **(A)** HLA-A, **(B)** -B and **(C)** -C. Red labels indicate the number of cases per box. Boxplots depict the median (horizontal line), mean (triangle), and first to third quartile (box); the highest and lowest values within 1.5× IQR (whiskers) and outliers (circles), respectively. *, p ≤ 0.05; ***, p ≤ 0.001; HLA, human leukocyte antigen; CSA, child-specific HLA antibody; IQR, interquartile range.

Snowflake score calculations considered the median solvent accessibility score (0.3717) in the panel of reference alleles as reported by (16). The interlocus-restricted locus-specific Snowflake scores in the PC were significantly higher in the CSA-positive compared to the CSA-negative groups for HLA-A, -B and -C (**Figure 1**), considering only locus-mismatched cases.

Within the pooled PC dataset, 566 HLA-mismatches were analyzed within a single model, corresponding to 121 child-specific antibodies. Correlation analysis revealed weak to moderate correlation between Snowflake and PIRCHE (0.36 ≤ r_s_ ≤ 0.49, p < 0.001) and a strong correlation with AllEps (r_s_ = 0.63, p < 0.001) as well as AbvEPS (r_s_ = 0.72, p < 0.001) (**Supplementary Figure 1**). Univariable logistic regression identified all considered models (except for PIRCHE-DRB3/4/5) as statistically significant. However, stepwise binomial multiple logistic regression suggested a minimal combination of Snowflake and PIRCHE presented by HLA-DP as independent contributors (**Table 2**).

**Table 2:** Univariable binomial logistic regression and minimal model created by stepwise binomial logistic regression of considered epitope matching algorithm scores and the development of child-specific HLA antibodies.

Algorithm	Univariable regression			Multiple logistic regression		
	Odds ratio	CI	Significance (p)	Odds ratio	CI	Significance (p)
PIRCHE-DRB1	1.40	1.06-1.88	0.020			
PIRCHE-DRB3/4/5	1.24	0.98-1.58	0.075			
PIRCHE-DQ	1.35	1.11-1.67	0.004			
PIRCHE-DP	1.46	1.15-1.86	0.002	1.25	0.98-1.62	0.079
AbvEPS	1.59	1.29-1.96	< 0.001			
AllEPS	1.52	1.24-1.88	< 0.001			
Snowflake	1.63	1.33-1.99	< 0.001	1.55	1.25-1.91	< 0.001

CI, 95% confidence interval.

**Figure 2:**
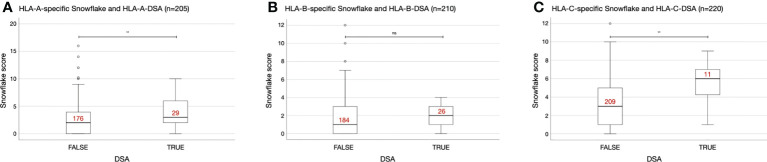
Interlocus-restricted Snowflake score distributions depending on HLA locus and presence of donor-specific antibodies (DSA). **(A)** HLA-A-specific Snowflake score in A-specific DSA negative/positive patients. **(B)** HLA-B-specific Snowflake score in B-specific DSA negative/positive patients. **(C)** HLA-C-specific Snowflake score in C-specific DSA negative/positive patients. Red labels indicate the number of cases per box. Boxplots depict the median (horizontal line), and first to third quartile (box); the highest and lowest values within 1.5× IQR (whiskers) and outliers (circles, rhombi), respectively. **, p ≤ 0.01; ns, p > 0.05; HLA, human leukocyte antigen; ISA, immunizer-specific antibodies; DSA, donor-specific HLA antibody; IQR, interquartile range.

Also in the KTC, statistically significantly higher HLA-A- and HLA-C-specific Snowflake scores were observed in cases with HLA-A- and HLA-C-specific DSA, respectively. Despite higher median HLA-B-specific Snowflake scores in HLA-B-specific DSA positive cases, the difference of distributions was not statistically significant (**Figure 2**).

In the pooled KTC dataset, 635 locus-specific epitope match scores were correlated with 66 DSA events. Correlation analysis indicated a strong correlation between PIRCHE presented by HLA-DRB1 and the remaining applied matching algorithms (0.64 ≤ r_s_ ≤ 0.71) and very strong correlations between AbvEPS, AllEPS and Snowflake (0.81 ≤ r_s_ ≤ 0.89, **Supplementary Table 1**). In univariable Cox regression, all four scores were significantly correlated with DSA (**Table 3**). Neither Cox multiple regression considering all four methods in a single model (**Supplementary Table 2**), nor pairwise Cox multiple regression (**Supplementary Table 3**) yielded statistically significant results.

**Table 3:** Cox regression analyses of PIRCHE-DRB1, AbvEPS, AllEPS and Snowflake predicting donor-specific antibodies in the KTC.

Algorithm	Cox regression		
	Odds ratio	CI	Significance (p)
PIRCHE-DRB1	1.65	1.24-2.19	0.001
AbvEPS	1.58	1.25-1.98	< 0.001
AllEPS	1.64	1.29-2.09	< 0.001
Snowflake	1.32	1.09-1.60	0.004

CI, 95% confidence interval.

To identify potential cross-reactivity between immunizing locus (e.g. HLA-A) and antibody target locus (e.g. HLA-B), for a subset of HLA-A-B-C mismatched cases of the PC (n=140), locus-specific Snowflake distributions were calculated (**Supplementary Table 4**). For HLA-A and -B CSA, multiple logistic regression suggests independent value of considering Snowflake from HLA-A and -B simultaneously. For CSA against HLA-C univariable regression does not find statistically significant correlation. As the supposed cross-reactivity may also be a proxy for T cell help due to variable dependency, PIRCHE-II scores were added to this regression analysis (**Table 4**). For the HLA-A and -B-specific models, indirect T cell epitopes derived from HLA-B mismatches were considered as independent contributors to developing CSA. Given the dependency of Snowflake and PIRCHE on the same locus, p values increase (i.e. Snowflake B and PIRCHE-DRB1-B). Counterintuitively, the model for HLA-A CSA considers PIRCHE from HLA-B. The model for HLA-B CSA however does not benefit from inclusion of PIRCHE derived from HLA-A but improves by inclusion of HLA-A Snowflake scores.

**Table 4:** Univariable binomial logistic regression and minimal model created by stepwise binomial logistic regression of considered Snowflake and PIRCHE scores of different loci and the development of child-specific HLA antibodies against a specific locus.

Algorithm	CSA	Univariable regression			Multiple logistic regression		
		Odds ratio	CI	Significance (p)	Odds ratio	CI	Significance (p)
Snowflake A	A	1.42	1.17-1.75	< 0.001	1.50	1.22-1.87	< 0.001
Snowflake B		1.19	0.94-1.50	0.154	1.23	0.94-1.61	0.122
Snowflake C		1.15	0.95-1.41	1.164			
PIRCHE-DRB1-A		1.56	0.95-2.71	0.093			
PIRCHE-DRB1-B		1.81	0.96-3.54	0.073	1.84	0.92-3.84	0.092
PIRCHE-DRB1-C		1.03	0.63-1.71	0.916			
Snowflake A	B	1.22	1.02-1.47	0.034	1.31	1.08-1.61	0.007
Snowflake B		1.36	1.09-1.71	0.008	1.38	1.07-1.78	0.013
Snowflake C		1.21	1.00-1.47	0.056			
PIRCHE-DRB1-A		1.01	0.66-1.58	0.963			
PIRCHE-DRB1-B		2.08	1.13-4.00	0.022	1.87	0.97-3.75	0.067
PIRCHE-DRB1-C		1.17	0.73-1.91	0.53			
Snowflake A	C	1.22	0.98-1.53	0.068	1.26	1.01-1.58	0.044
Snowflake B		1.18	0.89-1.54	0.241	1.23	0.93-1.62	0.142
Snowflake C		1.23	0.97-1.57	0.091			
PIRCHE-DRB1-A		0.86	0.51-1.49	0.58			
PIRCHE-DRB1-B		1.25	0.61-2.65	0.55			
PIRCHE-DRB1-C		1.48	0.81-2.91	0.222			

CI: 95% confidence interval, PIRCHE-DRB1-A: log-transformed HLA-A mismatch-derived allo-peptides presented by recipient DRB1, PIRCHE-DRB1-B: log-transformed HLA-B mismatch-derived allo-peptides presented by recipient DRB1, PIRCHE-DRB1-C: log-transformed HLA-C mismatch-derived allo-peptides presented by recipient DRB1.

## Discussion

Confirming our hypothesis, Snowflake scores were significantly higher in mismatched cases of both cohorts across HLA-A, -B and -C if they developed ISA against the respective locus (**Figures 1** and **2**, except for HLA-B in the KTC). By design, Snowflake considers HLA allele-matched pairs with a score of zero. Consequently, there is added value in prediction of ISA to classical HLA antigen matching by applying Snowflake matching. Previous reports on the extended KTC have shown correlations of epitope mismatch and DSA (20). Similarly, in the PC, correlation of epitope mismatch and CSA has been reported (11, 18, 19, 32). Correlation analyses revealed a moderate to strong correlation between all evaluated epitope matching approaches, which is in line with the literature (20, 33, 34). Notably, in both cohorts Spearman’s rho was highest between Snowflake and Eplets, confirming our second hypothesis. Both approaches aim on predicting HLA protein surface patches that are antibody accessible and thus potential targets for antibodies. Univariable regression analyses confirmed that all tested epitope matching strategies are significantly predictive of ISA (**Tables 2** and **3**). Multiple regression however is inconclusive about the independence of tested predictors. In the PC, the combination of Snowflake and PIRCHE-DP formed a minimal model with best CSA prediction (**Table 3**). In the KTC, the cohort size and number of events were insufficient to devise a combination of scores to independently predict DSA (**Supplementary Table 3**). Restricting the Snowflake algorithm to intralocus comparison also resulted in significant correlation with immunizer-specific antibodies but with reduced correlation to the reference epitope matching algorithms (data not shown).

It has been shown previously that the HLA protein surface varies between HLAs. HLA molecular matching algorithms applying locus- or class-wide maps of solvent-accessibility may thus over- or underestimate the proteins’ actual surface-residues. Consequently, the Snowflake algorithm has been proposed, considering surface-accessible amino acid positions defined per individual allele (16). This is the first study on the novel Snowflake algorithm to correlate HLA protein-specific surface-accessible amino acid mismatches and immunizer-specific antibody development. Our study features a back-to-back analysis of two cohorts with simultaneous analyses of reference epitope matching algorithms and the Snowflake algorithm allowing to evaluate overlap analyses between the different approaches.

However, there are also limiting factors that need to be considered. Contrary to the well-characterized and immunologically homogeneous PC, the KTC is immunologically much more diverse, with only intermediate-resolution HLA typings available, differing observation periods, varying donor-histocompatibility and immunosuppressive medication. Despite the advantage of epitope matching in kidney transplant cohorts (20, 35), age, non-adherence, comorbidities and several other factors thus confounding observations (36, 37). Although some of these confounders can be ruled out by backing the observations in the KTC by data from the PC, the immunological difference between organ transplantation and pregnancy have to be taken into consideration.

Reed et al. (38) have shown HLA sensitization being detectable already in the first trimester of pregnancy, yet the delivery has to be considered as an additional sensitizing event due to increased exposure to fetal cells. Thus, antibody levels may still increase after the sample collection period of up to four days post delivery. Unfortunately, in the PC dataset no further serum samples were collected prior and post delivery, to analyze kinetics of detected antibodies.

In the rather homogenous KTC, imputation has been applied to extend intermediate resolution typings to two-field typing levels. Although imputation is known to introduce inaccuracies into epitope analysis (39), the clinical impact of mispredictions seems limited for a vast majority of cases (28). Further studies examining kidney transplant recipients with equally comprehensive DSA follow-up but with two-field typing resolution of both the recipient and the donor are suggested to further support our findings and to rule out imputation-introduced bias. In addition, the Snowflake prediction pipeline is planned to be extended to also consider HLA Class II loci, which is challenging due to their heterodimeric structures.

Although solvent accessibility of antibody epitopes is necessary for antibody binding, it must be considered that other factors like molecular complementarity between epitope and binding site, size differences, physicochemical dissimilarity from self antigen, HLA expression levels and confirmation by T cell help impact the process. Consequently, the suggested Snowflake prediction pipeline may be further improved in the future by considering inclusion of these concepts.

Although the Snowflake prediction pipeline allows real-time analysis of new allele sequences, the surrounding Snowflake web service is as of now restricted to a specific IMGT version. Future versions of Snowflake may support upload of novel alleles.

Our analysis also revealed co-occurrence of cross-reactive elevated Snowflake scores (**Supplementary Table 4**, **Table 4**). In a multiple logistic regression of the PC, Snowflake scores of HLA-B mismatches were independently from Snowflake scores of HLA-A mismatches correlated with CSA against HLA-A (and for HLA-B respectively), despite HLA-A and -B having little overlap in surface-exposed amino acid configurations. It must be noted though, that the applied regression analysis of Snowflake quantities may not be capable of ruling out this phenomenon being an artifact of linkage disequilibrium or cross-locus T-cell help. To some extent, this is supported by selection of PIRCHE scores in multiple stepwise regression, yet for HLA-B-specific CSA, the Snowflake score against HLA-A remains a significant contributor. In order to confirm Snowflake scores inducing interlocus cross-reactivity that promotes antibody production and to dissect masking (potentially interlocus) effects of indirect T cell help, further studies are warranted.

Similar to the recently described HLA-EMMA matching algorithm (14), Snowflake predicts the number of solvent-accessible amino acid mismatches between donor and recipient. Opposed to HLA-EMMA however, each HLA’s surface is evaluated individually, rather than by locus. As indicated by (16), the individual amino acid configuration may alter the solvent-accessibility profile of HLA proteins.

Although the presence of DSA is known to be a major risk factor for graft rejection and loss of graft function (40–43) in all organ transplant domains, the Snowflake algorithm does not predict the clinical impact of each of the developed DSA. The applied epitope matching approaches - including Snowflake - do not weigh individual immunogenicity patterns. Similar to antigen mismatches varying in their immunogenicity, also epitope mismatches’ immunogenicity varies (11, 12, 19, 32). Although the current epitope matching strategies add prognostic value to classic antigen matching as implemented by many allocation organizations, further refinements of these approaches may specifically integrate epitope immunogenicity, antigenicity and hierarchy (44–46). Despite the current algorithms’ imperfections, simulation studies have shown the beneficial impact of currently available epitope matching algorithms in organ allocation (47, 48). Applied to a broad population, organ donor allocation supported by epitope matching may decrease overall frequency of immunological events that ultimately cause graft rejection. Consequently, improved average graft survival and fewer complications in post-transplant care may be hypothesized. In addition to further confirmatory allocation simulation studies and studies on individual patient level, evaluations of health economic benefits are desirable and will support the process of implementing and reimbursing such algorithms in national health systems.

In summary, we herein presented the first clinical evidence of the recently proposed Snowflake score correlating with ISA development. The strength of our study is the parallel analysis of two independent cohorts of pregnant women and patients who received kidney transplantation. In both cohorts, the number of HLA protein-specific surface-accessible amino acid mismatches (i.e. Snowflake score) was significantly associated with immunizer-specific antibody development, confirming the relevance of structural differences between HLA proteins for allorecognition. As hypothesized, a stronger correlation between Eplet (i.e. B cell epitopes) and Snowflake than between PIRCHE (i.e. indirect T cell epitopes) and Snowflake was observed. Further studies are warranted to elucidate the independence and contribution of Snowflake with respect to established epitope matching methods.

## Data availability statement

The data analyzed in this study is subject to the following licenses/restrictions: The data that support the findings of this study are available from the corresponding author upon reasonable request. Requests to access these datasets should be directed to Matthias Niemann, matthias.niemann@pirche.com.

## Ethics statement

The studies involving human participants were reviewed and approved by Ethikkommission beider Basel (EKBB) and Ethikkommission der Charité - Universitätsmedizin Berlin. The patients/participants provided their written informed consent to participate in this study.

## Author contributions

MN, YS, NL, FH, KB, GH, SS, BM, ES MN, NL and ES contributed to conception and design of the study. FH, KB, SS and GH provided retrospective cohort data for analysis. Data curation has been performed by YS, GH and MN. Formal analysis has been carried out by MN, BM and YS. MN wrote the first draft of the manuscript. All authors contributed to the article and approved the submitted version.

## Funding

This study is supported by the German Federal Ministry for Economic Affairs and Climate Action (grant 01MJ21002B).

## Conflict of interest

MN works for PIRCHE AG, which develops and operates the PIRCHE web service. PIRCHE AG and UMC Utrecht have filed a patent application on the prediction of an alloimmune response against allele-specific solvent-accessible amino acid mismatches. MN and ES are listed as inventors on this patent.

The remaining authors declare that the research was conducted in the absence of any commercial or financial relationships that could be construed as a potential conflict of interest.

## Publisher’s note

All claims expressed in this article are solely those of the authors and do not necessarily represent those of their affiliated organizations, or those of the publisher, the editors and the reviewers. Any product that may be evaluated in this article, or claim that may be made by its manufacturer, is not guaranteed or endorsed by the publisher.

## Abbreviations

CSA, child-specific antibodies; DSA, donor-specific antibodies; AbvEPS, interlocus-mismatched antibody-verified donor eplets; AllEPS, interlocus-mismatched donor eplets; ETKAS, Eurotransplant Kidney Allocation System; HLA, human leukocyte antigen; IQR, interquartile range; ISA, immunizer-specific antibodies; KTC, kidney transplant cohort; PC, pregnancy cohort; SAB, single HLA antigen beads; SD, standard deviation; SSO, sequence-specific oligonucleotide; SSP, sequence-specific primer.
