# Supplementary material for: Snowflake epitope matching correlates with child-specific antibodies during pregnancy and donor-specific antibodies after kidney transplantation
Source: Front Immunol. 2022 Oct 28;13:1005601. doi: 10.3389/fimmu.2022.1005601 (PMC9649433; doi:10.3389/fimmu.2022.1005601)
Supplement: Supplementary file 1 [file DataSheet_1.docx]

***Supplementary Material***


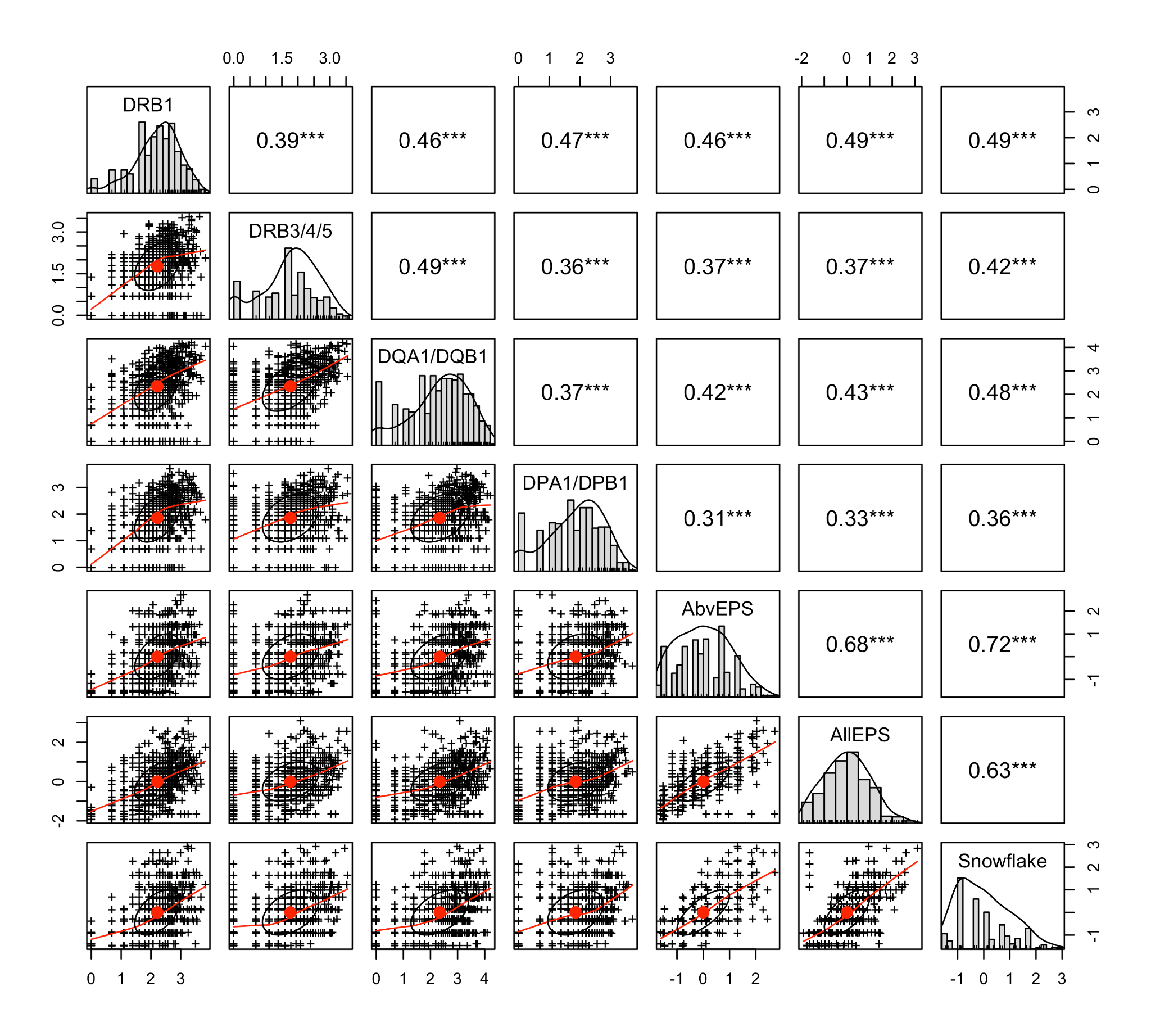


Supplementary Fig 1: Correlation scatter matrix of scaled (AbvEPS, AllEPS, Snowflake) and logarithmic (PIRCHE-DRB1, PIRCHE-DRB3/4/5, PIRCHE-DQA1/DQB1, PIRCHE-DPA1/DPB1) epitope match scores in the PC. Numbers indicate Spearman’s rho (r_s_). ***: p < 0.001.

Supplementary Table 1: Correlation matrix of scaled (AbvEPS, AllEPS, Snowflake) and logarithmic (PIRCHE-DRB1) epitope match scores in the KTC. Numbers indicate Spearman’s rho (r_s_). **: p < 0.01.

| Spearman’s rho | PIRCHE-DRB1 | AbvEPS | AllEPS |
| --- | --- | --- | --- |
| AbvEPS | 0.638** | - | - |
| AllEPS | 0.710** | 0.886** | - |
| Snowflake | 0.636** | 0.814** | 0.822** |

Supplementary Table 2: Multiple Cox regression of PIRCHE-DRB1, AbvEPS, AllEPS and Snowflake. CI, 95% confidence interval.

| Algorithm | Pairwise Cox multiple regression | | |
| --- | --- | --- | --- |
|  | Odds ratio | CI | Significance (p) |
| PIRCHE-DRB1 | 1.32 | 0.92-1.90 | 0.135 |
| AbvEPS | 1.23 | 0.77-1.97 | 0.387 |
| AllEPS | 1.21 | 0.70-2.07 | 0.497 |
| Snowflake | 0.94 | 0.68-1.28 | 0.683 |

Supplementary Table 3: Pairwise Cox multiple regression of (1) PIRCHE-DRB1 and AbvEPS, (2) PIRCHE-DRB1 and Snowflake, (3) PIRCHE-DRB1 and AllEPS, (4) AbvEPS and Snowflake, (5) AbvEPS and AllEPS and (6) AllEPS and Snowflake predicting dnDSA. CI, 95% confidence interval.

| Pair | Algorithm | Pairwise Cox multiple regression | | |
| --- | --- | --- | --- | --- |
|  |  | Odds ratio | CI | Significance (p) |
| 1 | PIRCHE-DRB1 | 1.37 | 0.98-1.91 | 0.064 |
|  | AbvEPS | 1.34 | 1.01-1.78 | 0.046 |
| 2 | PIRCHE-DRB1 | 1.55 | 1.13-2.13 | 0.007 |
|  | Snowflake | 1.11 | 0.87-1.41 | 0.400 |
| 3 | PIRCHE-DRB1 | 1.31 | 0.91-1.88 | 0.141 |
|  | AllEPS | 1.39 | 1.00-1.93 | 0.053 |
| 4 | AbvEPS | 1.56 | 1.14-2.13 | 0.005 |
|  | Snowflake | 1.01 | 0.76-1.35 | 0.924 |
| 5 | AbvEPS | 1.21 | 0.76-1.91 | 0.429 |
|  | AllEPS | 1.39 | 0.86-2.25 | 0.185 |
| 6 | AllEPS | 1.66 | 1.19-2.32 | 0.003 |
|  | Snowflake | 0.99 | 0.73-1.34 | 0.927 |

Supplementary Table 4: Univariable binomial logistic regression and minimal model created by stepwise binomial logistic regression of considered Snowflake scores of different loci and the development of child-specific HLA antibodies against a specific locus. CI: 95% confidence interval

|  | CSA | Univariable regression | | | Multiple logistic regression | | |
| --- | --- | --- | --- | --- | --- | --- | --- |
| Algorithm |  | Odds ratio | CI | Significance (p) | Odds ratio | CI | Significance (p) |
| Snowflake A | A | 1.42 | 1.17-1.75 | < 0.001 | 1.48 | 1.21-1.84 | < 0.001 |
| Snowflake B |  | 1.19 | 0.94-1.50 | 0.154 | 1.30 | 1.01-1.69 | 0.042 |
| Snowflake C |  | 1.15 | 0.94-1.41 | 0.164 |  |  |  |
| Snowflake A | B | 1.22 | 1.02-1.47 | 0.034 | 1.30 | 1.07-1.59 | 0.009 |
| Snowflake B |  | 1.36 | 1.09-1.71 | 0.008 | 1.45 | 1.15-1.87 | 0.002 |
| Snowflake C |  | 1.21 | 1.00-1.47 | 0.056 |  |  |  |
| Snowflake A | C | 1.23 | 0.98-1.53 | 0.068 | 1.26 | 1.00-1.58 | 0.044 |
| Snowflake B |  | 1.18 | 0.89-1.54 | 0.241 | 1.23 | 0.93-1.62 | 0.14 |
| Snowflake C |  | 1.23 | 0.97-1.57 | 0.091 |  |  |  |
